# Fetal Speckle Tracking Technology for Critical Aortic Stenosis: Advancing Through Innovation

**DOI:** 10.3390/diagnostics15202591

**Published:** 2025-10-14

**Authors:** Julia Murlewska, Sławomir Witkowski, Iwona Strzelecka, Maria Respondek-Liberska

**Affiliations:** 1 Department of Prenatal Cardiology, Polish Mother’s Memorial Hospital Research Institute, 93-338 Lodz, Poland; juliamurlewska.jm@gmail.com (J.M.);; 2 Medical Faculty, Ludwik Rydygier Collegium Medicum in Bydgoszcz, 85-067 Bydgoszcz, Poland; 3 Department of Diagnoses and Prevention of Fetal Malformations of Medical, University of Lodz, 90-136 Lodz, Poland

**Keywords:** aortic stenosis, fractional area change, ejection fraction, global strain, fetal heart failure, fetal echocardiography, fetal speckle tracking

## Abstract

**Background and Clinical Significance**: This article explores the application of fetal speckle tracking technology in evaluating critical aortic stenosis (AS) in fetuses, highlighting its potential for predicting neonatal outcomes. **Case Presentation**: We present two cases of fetuses diagnosed with critical AS and associated complications at late gestation. Case 1 demonstrated preserved left ventricular function, as indicated by favorable global strain (GS), fractional area change (FAC), and ejection fraction (EF) values, despite critical conditions. This infant underwent successful postnatal interventions and was discharged after an extended NICU stay. In contrast, Case 2 exhibited severely compromised left ventricular function with significantly reduced GS, FAC, and EF parameters, leading to a tragic outcome despite intensive management. **Conclusions**: Our findings suggest that innovative echocardiographic parameters such as GS, FAC, and EF for the left ventricle are crucial in prognostic evaluations for fetuses with critical AS. The study underscores the importance of advancements in fetal cardiology and the need for further research to enhance prognostic assessments and improve clinical outcomes in affected neonates.

## 1. Background

Echocardiographic prognostic markers for newborns with critical aortic stenosis include hemodynamic and morphometric factors that can help predict survival and guide treatment decisions.

Some of the markers studied include hemodynamic measurements of flow in the aorta (ascending, transverse, and descending); flow measurements across the ductus arteriosus, aortic valve, and mitral valve; and morphometric measurements of the left heart structures [1,2,3].

New fetal HQ technology (fetal speckle tracking) allows for the assessment of the GL global strain, FAC (fractional area change), and EF (ejection fraction) in mid-to-late pregnancy to predict the overall risk overall neonatal complications [1,4], which we would like to present in the following parts of the article based on two cases of fetuses with critical aortic stenosis.

## 2. Case Presentation

Case 1: This case represents a complex prenatal cardiac condition characterized by critical aortic stenosis (AS), mitral valve stenosis (MS), and restrictive foramen ovale (FO) with accompanying ascites, diagnosed at 35 weeks and 4 days of gestation. Gestational age was established by the last menstrual period, and serial ultrasounds confirmed consistency of follow-up. The estimated fetal weight (EFW) was 4125 g at 35 weeks, which contrasted with the actual birth weight of 3300 g. This discrepancy was likely related to abdominal circumference increase due to ascites and hepatomegaly versus limitations in gestational age assessment. Screening for gestational diabetes was performed and was negative. No other structural malformations were identified outside the cardiac findings. Doppler echocardiography revealed severe narrowing of the aortic valve and compromised cardiac function, as evidenced by a low cardiovascular profile score (CVPS), together with cardiomegaly, tricuspid regurgitation (TR), and mitral regurgitation (MR). In the fetal HQ study, some functional parameters for the left ventricle, such as global strain (GS −38.8%), fractional area change (FAC 47.7%), and ejection fraction (EF 39.9%), were relatively preserved. Maternal intravenous therapy was initiated to deliver digoxin, corticosteroids, and antibiotics transplacentally; that is, the medications were administered to the mother with the intent of crossing the placenta and acting on the fetus indirectly.

Three days later, worsening hemodynamic compromise led to an emergency cesarean section due to non-reassuring fetal status. The deterioration in fetal hemodynamics was reflected by a worsening cardiovascular profile score (CVPS), increased tricuspid and mitral regurgitation, progressive cardiomegaly, and abnormal venous Doppler waveforms, including reversal flow in the ductus venosus.

Postnatal management in the neonatal intensive care unit (NICU) included a Rashkind procedure and balloon aortic valvuloplasty on the 8th day of life, followed by a Norwood procedure on the 12th day. Norwood surgery was performed because the left ventricular function was inadequate for biventricular repair. Operative details included the following: duration of cardiopulmonary bypass—120 min; cross-clamp time—40 min; type of shunt used—modified Blalock–Taussig (BT) shunt; and cooling strategy—deep hypothermic circulatory arrest (DHCA). The infant required prolonged hospitalization (231 days), with complications including necrotizing enterocolitis and ascites, but was ultimately discharged in good condition (all the details are presented in Table 1).

**Figure 1:**
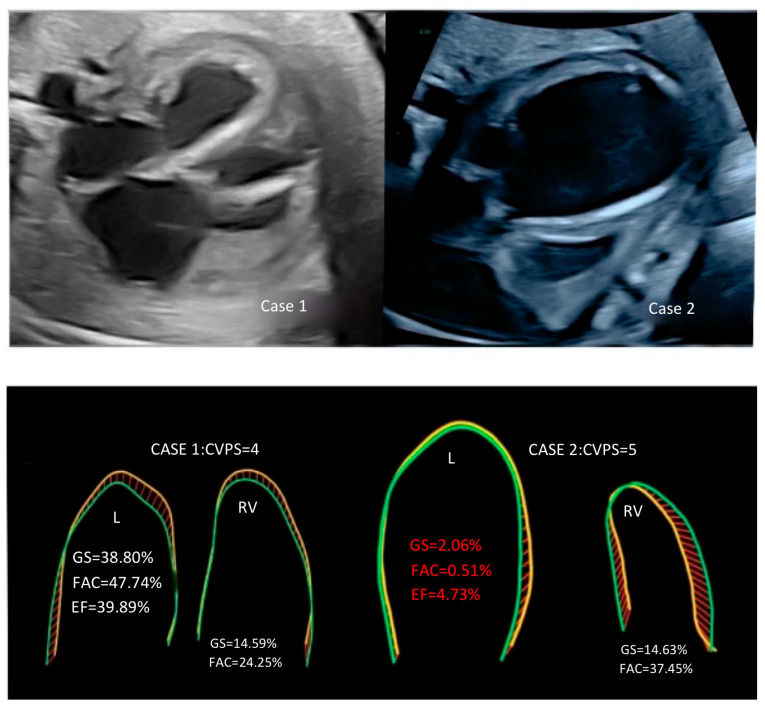
Four-chamber view in case nr 1 and case nr 2 with critical aortic stenosis, with schematic presentation of functional parameters for the left and right ventricles in fetuses at 35 weeks of pregnancy, with significantly reduced parameters: GS—global strain; FAC—fractional area change; and EF—ejection for left akinetic. Dilated left ventricle with endocardial fibroelastosis for case 2. End-diastole and end-systole are defined in different colors.

Case 2: Gestational age at diagnosis was 35 weeks and 3 days, confirmed by last menstrual period. The case described involves a similar complex and severe cardiac condition, diagnosed prenatally. Critical aortic and mitral valve stenosis, identified late in the pregnancy, presented significant challenges for fetal intervention. The absence of eligibility for balloon aortic valvuloplasty (BAV) surgery further complicated the clinical decision-making process. The echocardiographic findings, including an extremely enlarged left ventricle with fibroelastosis (Figure 1) and severely reduced functional parameters such as global strain, fractional area change, and ejection fraction, indicated a compromised cardiac function. The CVPS—cardiovascular profile score, which assigns points based on the severity of various cardiac and associated findings—of only 5 points reflected the critical nature of the condition. Despite the implementation of intensive prenatal pharmacological transplacental therapy (i. v. digoxin, steroids, antibiotics) and postnatal therapy with ECMO—extracorporeal membrane oxygenation—and subsequent surgical interventions post-birth, including the Rashkind procedure, BAV, and the Norwood procedure, the infant’s condition remained critical, ultimately leading to a tragic outcome and her death on the 28th day of life (all the details are provided in Table 1). The newborn was delivered at 36 + 1 weeks (late preterm). Maternal screening for gestational diabetes was negative, and no additional extracardiac malformations were detected. Cesarean section was performed due to worsening fetal heart failure with abnormal Doppler findings.

Postnatally, the infant required extracorporeal membrane oxygenation (ECMO) beginning in the first 24 h of life because of refractory low cardiac output. A Rashkind atrial septostomy and balloon aortic valvuloplasty were performed on day 6, followed by a Norwood procedure on the same day. Operative parameters included a cardiopulmonary bypass time of 120 min, an aortic cross-clamp time of 40 min, use of a modified Blalock–Taussig, and cooling to 20 °C. Postoperative complications, including severe ventricular dysfunction, arrhythmias, renal failure, and bleeding, appeared within the first 48 h after surgery. Despite maximal supportive therapy, the infant’s condition deteriorated, and she died on the 28th day of life.

## 3. Discussion

In a fetus with critical aortic stenosis, several biomechanical changes occur in the left ventricle of the heart [6]. These changes include elevated left ventricular (LV) pressures due to obstruction in the left ventricular outflow tract [1,7,8], decreased EF [%] [4,9], and diminished global systolic and diastolic function, which may be measured by the classical Tei index [10] or FAC [%] using the speckle tracking technique [4,9]. The Tei index—also known as the Myocardial Performance Index (MPI)—includes both systolic and diastolic time intervals to evaluate cardiac performance [10].

We presented two cases of fetuses with late prenatal diagnosis of critical aortic valve stenosis and fetal heart failure (FHF) in the 35th week of pregnancy who did not qualify for prenatal cardiac balloon surgery. Some important echocardiographic parameters in a fetus with critical aortic stenosis include aortic parameters, pulmonary valve annulus sizes, and Doppler interrogation [1,6,7,8], which are all presented in Table 1, and in both cases, we noted significant aortic stenosis with significantly accelerated PSV—peak systolic velocity [cm/s]—with the assessment of pericardial effusion (which was observed in case 1), ascites (which was observed in case 2) with cardiomegaly, and endocardial LV fibroelastosis being observed in each case, with significant left ventricular dilation in case 2. Retrograde flow in the aortic arch, mitral stenosis and regurgitation, and left to right flow across the restricted foramen ovale were also seen in both cases, with accelerated and reversed pulmonary vein flows. The survival of a newborn with AS depends on the function of the left ventricle, which is crucial for pumping oxygenated blood to the body and ensuring proper circulation. Studies have shown that Fetal Strain measurements are more reliable for the left ventricle than for the right ventricle in assessing EF and FAC [11,12]. FAC is a measurement that provides an estimate of the global systolic function of the ventricles. It calculates the percentage of area change within chambers between the diastole (relaxation phase) and systole (contraction phase). The value provided is a percentage. The difference between end-diastolic and end-systolic areas of the ventricles divided by the end-diastolic area is the FAC. The normal value of the FAC is typically 35% or higher. The EF is a measurement, expressed as a percentage, of how much blood the left ventricle pumps out with each contraction. An EF below 40 percent might be evidence of fetal heart failure [4,9,12]. The normal values of the Tei index can vary slightly depending on the specific measurement methods and populations studied. However, in general, the normal range for the Tei index is typically considered to be around 0.39 ± 0.05 [10]. In both our cases of fetuses with critical aortic stenosis, MPI indices were higher, but the GS, FAC, and EF for the left ventricle were much more sensitive for differentiating both cases, and those parameters should be used to analyze and monitor fetuses with AS. The right atrium is generally considered more important in a fetus than the left atrium due to its role in receiving deoxygenated blood from the body and directing blood flow through the foramen ovale to the left atrium, allowing oxygenated blood from the placenta to bypass the non-functioning lungs. In our cases, we observed comparable deteriorated fetal HQ parameters for the right ventricle and left and right atrium (Table 1). Despite the occurrence of abnormal flows in the pulmonary veins in both cases, only fetus number 2 died postnatally, probably due to the presence of much worse functional parameters in fetal HQ for the left ventricle (GS, EF, FAC) (Figure 1).

The main limitations of this report relate to the echocardiographic methodology rather than to the individual cases. The image quality, frame rates, and fetal position can substantially affect the accuracy and reproducibility of speckle tracking measurements. In our two cases, the frame rates were within the recommended range, and the image quality was judged to be adequate, but assessments were performed late in gestation, which may have limited optimal tracking. All analyses were conducted and verified by experienced fetal cardiologists to minimize observer variability. Furthermore, normative reference data for fetal global longitudinal strain (GLS) and strain rate (GLSR) are still limited, which restricts the generalizability of these findings and underlines the need for larger prospective studies.

## 4. Conclusions

The advancements in fetal speckle tracking echocardiography have provided valuable insights into the prognostic evaluation of critical aortic valve stenosis in fetuses. Parameters such as the global strain (GS), fractional area change (FAC), and ejection fraction (EF) of LV measures could be investigated as potential tools for stratifying risk factors in fetuses with critical aortic stenosis. This highlights the potential of speckle tracking technology in assessing the postnatal outcome, suggesting a need for further research in this area. Our pioneering findings in this field underscore the importance of continuous innovation and exploration in fetal cardiology to improve prognostic assessments and outcomes.

## Figures and Tables

**Table 1:** The table presents a detailed comparison of classic echocardiographic parameters, as well as innovative parameters in fetal HQ technology, as well as the characteristics of the postnatal state for 2 described cases with critical prenatally diagnosed aortic valve stenosis. Global strain values are shown with a negative sign to indicate myocardial shortening, consistent with the convention in speckle tracking analysis. Despite a normal cerebroplacental ratio (CPR) in case 1, the fetus exhibited other signs of decompensation, including ascites, cardiomegaly, abnormal venous flow, and significant valvular regurgitation. This highlights that a normal CPR alone may not be sufficient to rule out advanced fetal heart failure in cases of critical aortic stenosis.

Critical Aortic Stenosis, FHF, Restricted FO, Prenatal Oxygen Therapy, Steroid Therapy, Antibiotic Therapy, and Glycosides for Cases 1 + 2	Case 1	Case 2
Gestational age at prenatal diagnosis, established at last menstrual period	35 w 4 d	35 w 3 d
EFW [g] established at Hadlock [5]	4125	2430
CVPS points	4	5
HA/CA	0.6	0.6
AFI [cm]	23	20
UMB PI—umbilical cord pulsatility index	0.52	2.37
MCA PI—middle cerebral artery pulsatility index	0.92	1.4
DV—ductus venosus reversal flow	present	absent
MCA PSV [cm/s]—middle cerebral artery peak systolic velocity	50	48
Tei LV—Tei index for left ventricle	0.7	0.77
Tei RV—Tei index for right ventricle	0.7	0.7
AoV diameter [mm]— aortic valve diameter	3	4.8
AoV PSV [cm/s]— aortic valve peak systolic velocity	300	260
PAV diameter [mm]—pulmonary artery valve diameter	9.7	11
PAV PSV [cm/s]—pulmonary artery valve peak systolic velocity	98	65.3
PV PSV [cm/s]—pulmonary vein peak systolic velocity	53	50
M—mode EF [%]	40	5
MV/TV annular ratio	0.9	0.5
AoV/PAV annular ratio	0.8	0.4
LV/RV mid-cavity ratio	1.0	0.4
LV/RA long-axis ratio	1.1	0.6
Global strain LV [%]	−38.80	−2.06
Frac. area change LV [%]—fractional area change	47.74	−0.51
EF LV [%]—ejection fraction for left ventricle	39.89	4.73
Global strain RV [%]	−14.59	−14.63
Frac. area change RV [%]—fractional area change	24.25	37.45
Global strain RA [%]	−17.98	−32.62
Frac. area change RA [%]—fractional area change	26.09	44.28
Global strain LA [%]	−3.14	−2.01
Frac. area change LA [%]—fractional area change	3.12	2.12
Time of delivery	35 w 7 d	36 w 1 d
Newborn weight	3300 g	2750 g
Newborn Apgar scores	7,7,7	7,8,8
Newborn gender	boy	girl
Type of delivery	CS	CS
Balloon atrial septostomy	8th day of life	6th day of life
BAV—balloon aortic valvuloplasty	8th day of life	6th day of life
Norwood procedure	12th day of life	6th day of life
Follow-up	231 days of hospitalization: NEC—necrotizing enterocolitis, ascites	ECMO, death on the 28th day of life

Abbreviations: AFI, amniotic fluid index; AoV, aortic valve; BAV, balloon aortic valvuloplasty; CS, cesarean section; CVPS, cardiovascular profile score; DV, ductus venosus; EF, ejection fraction; EF LV, ejection fraction of the left ventricle; FO, foramen ovale; HA/CA, heart-area-to-chest-area ratio; LA, left atrium; LV, left ventricle; MCA PI, middle cerebral artery pulsatility index; MCA PSV, middle cerebral artery peak systolic velocity; PAV, pulmonary artery valve; PSV, peak systolic velocity; PV, pulmonary vein; RA, right atrium; RV, right ventricle.

## Data Availability

The original contributions presented in this study are included in the article. Further inquiries can be directed to the corresponding authors.
